# ADAPTATEA Program: Humanizing Healthcare for ASD Patients in Their Referral Hospital

**DOI:** 10.1192/j.eurpsy.2025.1823

**Published:** 2025-08-26

**Authors:** L. Garcia Murillo, A. Cañuelo Marquez, A. Pascual, J. Sanchez Cerezo, R. Paricio, A. Guzman, M. Morales, I. Palanca

**Affiliations:** 1 Psiquiatría, Hospital Universitario Puerta de Hierro, Majadahonda, Spain

## Abstract

**Introduction:**

Autism Spectrum Disorder (ASD) is a neurodevelopmental disorder with increasing prevalence (1 in 36 children, CDC 2023), associated with difficulties in social interaction and repetitive behaviors. These characteristics make it challenging for individuals with ASD to access quality healthcare. Additionally, people with ASD experience a higher number of underlying and intercurrent comorbidities compared to neurotypical individuals.

In Spain, we have a public healthcare system intended to provide universal health coverage for all citizens. In 2009, the AMITEA program was created in central Madrid to address healthcare needs for 6,000,000 people (Parellada et al., Eur Psychiatry 2013; 28(2):102-109). However, despite this, patients in our hospital, located in Majadahonda on the outskirts of Madrid, continued to face difficulties accessing healthcare. Main challenges include leaving familiar surroundings and healthcare professionals, long waiting times, difficulties understanding the public healthcare system, and lack of coordination between services. We realized that, even with the program, our patients were still not receiving adequate care.

**Objectives:**

- To create an integrated program in our hospital that addresses the most common health issues for individuals with ASD.
- To provide ASD patients with healthcare attention as similar as possible to that of neurotypical patients within their referral hospital.

**Methods:**

Given the difficulties our patients faced, professionals from Child Psychiatry, Neuropediatrics, Neurophysiology, and the Pediatric ER formed a network in 2018 to improve healthcare for ASD patients in our hospital. Child Psychiatry acted as a link between services. We held group meetings with department leaders to develop the program in each service. Neurophysiology was the first to fully implement it (Mayoral-Fernandez et al., Metas Enferm 2022; 25(5):70-8).

**Results:**

Thanks to our efforts and support from hospital management, a part-time nurse joined the program in October 2023. With her help, we adapted medical procedures using Alternative Communication systems, trained and advised staff, adjusted hospital spaces, and maintained continuous coordination with professionals and families, humanizing care at our hospital.

By January 2024, our network included 17 services and over 50 professionals. With group support, each service developed its own level of adaptation. Professional satisfaction increased, and specialist coordination improved.

**Conclusions:**

Adaptations like those implemented through ADAPTATEA improve the healthcare experience for ASD individuals at their referral hospitals, highlighting the need for more programs of this kind.

**Disclosure of Interest:**

None Declared

